# Study of an Organic Binder of Cold-Bonded Briquettes with Two Different Iron Bearing Materials

**DOI:** 10.3390/ma14112952

**Published:** 2021-05-30

**Authors:** Ying Li, Huiting Chen, Abourehab Hammam, Han Wei, Hao Nie, Weitian Ding, Mamdouh Omran, Lixiang Yan, Yaowei Yu

**Affiliations:** 1 State Key Laboratory of Advanced Special Steel, Shanghai Key Laboratory of Advanced Ferrometallurgy, School of Materials Science and Engineering, Shanghai University, 99 Shangda Rd, Shanghai 200444, China; yingli@shu.edu.cn (Y.L.); huitingchen@shu.edu.cn (H.C.); a.e.hammam@hotmail.com (A.H.); weihan@shu.edu.cn (H.W.); niehao@shu.edu.cn (H.N.); dingweitian@shu.edu.cn (W.D.); mamdouh.omran@oulu.fi (M.O.); 2 Central Metallurgical Research and Development Institute (CMRDI), P.O. Box 87, Helwan, 11421 Cairo, Egypt; 3 Process Metallurgy Research Unit, University of Oulu, Pentti Kaiteran Katu 1, 90014 Oulu, Finland; 4 Chongqing Zhenyan Energy Saving and Environmental Protection Technology Co., Ltd., 3, 10 Yueguang Village, Xin Street, Chongqing 400084, China; li_li_li9999@163.com

**Keywords:** iron bearing materials, cold-bonded briquette, organic binder, bonding mechanism

## Abstract

The aim of this study was to investigate the properties of an organic binder used in cold-bonded briquettes (CBBs) prepared from two different iron bearing materials. The applied binder is a type of starch as indicated by chemical analysis, iodine-starch staining and Fourier transform infrared analyses. Thermogravimetric differential scanning calorimetry showed that the binder pyrolysis undergoes four stages: moisture desorption, ash volatilization, pyrolysis of organic matter and decomposition of materials with high activation energy. The difference between the dry and heat-treated samples during the macroscopic failure process is the instability propagation of the crack. The CBB shows a low decrepitation index at 700 °C. The returned fines of CBBs used with the organic binder were applied in two blast furnaces. The industrial trials showed that the CBBs do not influence the performance of the blast furnace and can reduce the fuel consumption rate. The curing rate of the binder decreases, and the growth rate of compressive strength decreases during the curing process. Iron ore particles are bonded together and exist in the form of aggregation after mixing with water and binder. The edges and corners of the particles become blurred, and the original surfaces of the particles are covered with binder film, the surface of which is covered with fine particles. The multi-branched structure of amylopectin provides omnibearing adhesion sites, thus forming binder agglomeration and film leading to a strong adhesion between binder and iron ore particles. Binder film and binder agglomeration work together to make the CBB perform well.

## 1. Introduction

Briquetting is an important process that is used to recycle and utilize the fine particles of steel plants as a product that provides suitable feed for metallurgical furnaces [1,2,3,4,5,6]. The briquetting process avoids heating up, softening, and melting the fine particles, which can save a lot of fossil energy and decrease the environmental pollution. A cold-bonded briquette (CBB) is the product of the briquetting process. The charging of the CBBs in a blast furnace depends on the physical and mechanical properties of the briquettes [1,7,8].

The quality of CBBs and their properties are dependent on the binder. Furthermore, the binder determines whether the quality of the CBB can directly charge in the furnaces. Various kinds of binders such as pitch, molasses, starch, bentonite, sodium silicate and cement, or mixtures of these, have been used as binders [1,9,10]. For example, EI-Hussiny et al. [1]. used pitch as a binder for the mixture of mill scale and blast furnace flue dust. Cui et al. [9] used 2% bentonite as a binder for the mixture of blast furnace dust, converter dust, electric arc furnace dust and converter sludge. Kumar et al. [7] investigated molasses, starch and sodium silicate as binders for the mixture of several types of dust and mill scale. In the previous works, many types of binders have been used for different raw materials, and each binder has different effects on the quality of the CBB. Various investigations [6,8,10,11,12,13] have studied the strength of the briquette and bonding mechanism with the binder. Unfortunately, due to commercial competition, the physical and chemical properties of commercial binder are vague. However, commercial binder usually has better performance; therefore, it is important to determine these properties in order to develop new binders.

In this work, the chemical and the thermal properties of commercial binder with good performance were firstly studied. Then, the relationship between CBB strength and the binder was investigated and compared with different raw material of return fines and the other iron ore fines. Finally, the bonding mechanism of the binder was also studied. The outcome from this study will provide information on how to develop a new binder, study the properties of CBBs and understand the bonding mechanisms of binder.

## 2. Materials and Methods

### 2.1. Materials

#### 2.1.1. Iron Ore Raw Material

**Figure 1:**
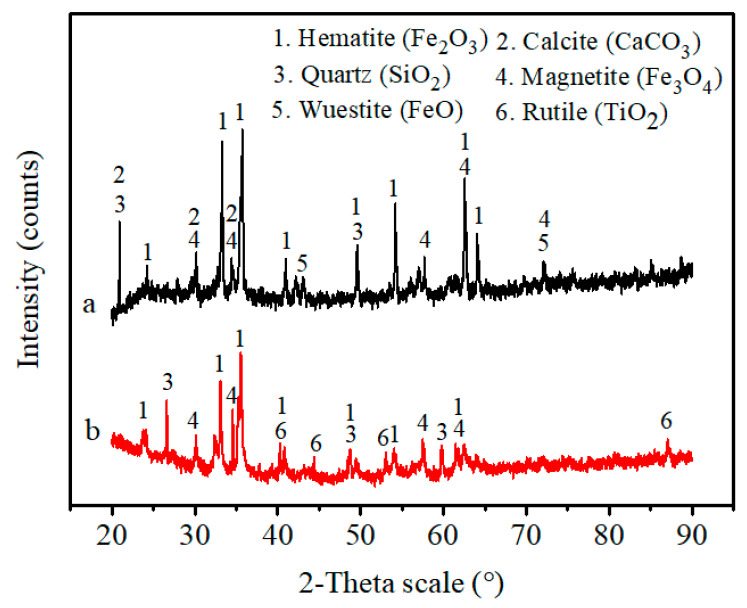
X-ray diffraction patterns for (**a**) sample A, (**b**) sample B.

Two types of iron bearing materials in this work came from Gansu Province, China, and they were labelled as samples A and B. Sample A is return fines of sinter generated in the sintering process; sample B is iron ore fines containing titanium, which could decrease the cost of protecting blast furnace lining. Return fines of sinter and iron ore fines containing titanium are usually utilized with the sintering process. A CBB avoids heating up, softening, and melting the fine particles, which can save a lot of fossil energy and manpower. Size ranges of samples A and B are 0.5–5 mm and less than 0.5 mm, respectively. Sample A has only less than 20 wt.% of fine (<0.5 mm). The detailed particle size distribution of samples A and B is shown in Table 1. The mineralogical composition was measured using X-ray diffraction (XRD). The measurements were taken using a Rigaku diffractometer (Ultima IV, Rigaku, Tokyo, Japan) with CuKa radiation, an operating voltage of 35 kV and an anode current of 20 mA. The measurement used a step size of 0.02° 2θ and step time of 1 s. Chemical compositions of samples A and B were detected using an X-ray fluorescence (XRF) spectrometer (XRF-1800, Shimadzu, Tokyo, Japan), and total C was measured with a carbon and sulfur analyzer (CS600, LECO, San Jose, MI, USA). Figure 1 and Table 2 show the XRD patterns and chemical analysis, respectively, of iron ore used in the tests. 

#### 2.1.2. Binder

**Figure 2:**
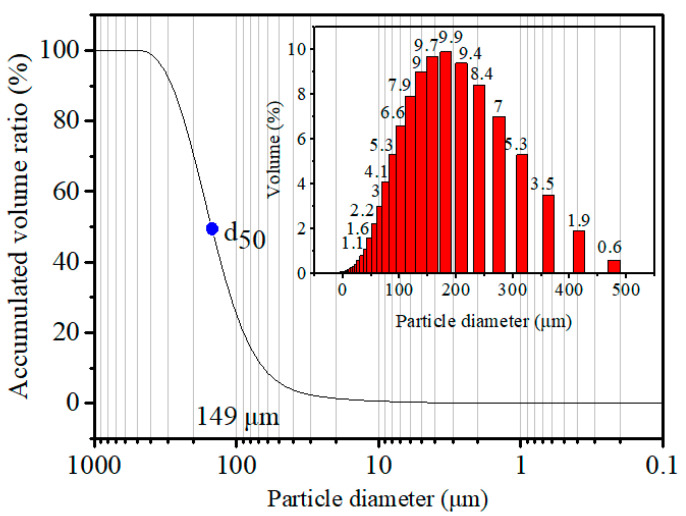
Size distribution of the binder.

A commercial binder with good performance during the briquetting process was selected. Size distribution of the binder was obtained by using particulate size description analyzer (PSDA, Malvern Mastersizer 2000, Malvern, UK), and the results are shown in Figure 2. It was found that median particle size for the binder was 149 μm, while nearly 50% of binder particles were in the range of 138 to 240 μm. In order to judge whether the binder contains inorganic elements, the binder was heated up to 500 °C for 10 min in a muffle furnace, then the binder ash was collected and analyzed to obtain the composition. The elements of the binder were detected using an organic element analyzer (OEA, Vario Micro cube, Elementar, Hanau, Germany). The elements of binder ash were determined by the XRF analysis. 

### 2.2. Experimental

#### 2.2.1. Binder Characterizations 

The binder structure was analyzed by Fourier transform infrared (FTIR) spectrometry and thermogravimetric differential scanning calorimetry (TG-DSC). The binder was measured on a Nicolet 380 FTIR spectrometer (Nicolet 380, Thermo Fisher Scientific, Waltham, MA, USA) using the KBr disc technique. For FTIR measurement, 1 mg binder was mixed with 100 mg anhydrous KBr and was then compressed into thin disk-shaped pellets. The spectra were measured with a resolution of 4 cm^−1^ between a wave number range of 4000 to 400 cm^−1^. The thermal analysis of the binder was carried out using TG-DSC (STA449F3, Netzsch, Selb, Germany). The type of crucible of the TG/DSC pan was Al_2_O_3_. The sample chamber was purged with nitrogen gas at a rate of 30 mL/min. TG and DSC analyses were performed with a heating rate of 10 °C/min in the temperature range of 28 to 1200 °C. Scanning electron microscopy (SEM, Nova NanoSEM 450, FEI, Columbia, MD, USA) was used to observe the microstructure of binder particles and to gain further knowledge of the iron ore particle structure and morphology after bonding with the binder.

#### 2.2.2. Preparation of Mixtures and Briquettes 

**Figure 3:**
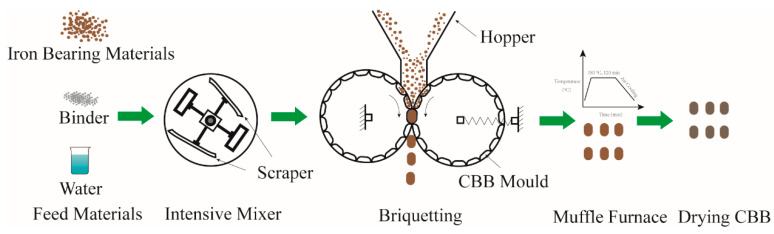
Diagram showing the process of preparing the CBBs.

The mixtures containing 88 wt.% iron ore, 3 wt.% binder and 9 wt.% water were obtained by sufficient mixing. A small amount of the mixtures was used to study the bonding mechanism by observing the functional group, phase composition, thermal behavior and microstructure. The remainder of the mixtures was used to prepare the briquette. The spherical-shape briquettes in dimensions of 40 mm × 40 mm × 20 mm (L × W × H) and about 45 g in weight were produced by a twin-roller machine with pressure of 500 kg/cm^2^. CBBs of samples A and B were labelled as samples AC and BC, respectively. The wet CBBs were dried in a muffle furnace with air as heated medium at 180 °C for 2 h. The process of preparing the CBBs is shown in Figure 3. 

#### 2.2.3. Testing of Mechanical Strength and Decrepitation Index of CBB

The drop strength test was carried out by dropping a CBB from a height of 1 m onto a 10 mm-thick steel plate until its breakage. Drop strength experiments were repeated 10 times. Drop strength values are the average of 10 tests. The compressive strength was measured on a universal testing machine (UTM, C45.305, MTS, Eden Prairie, MN, USA). According to the standard ISO 8371, the decrepitation index of the CBBs was measured at 700 °C, and then the mass of the broken briquettes was weighed in each size interval. In the standard test, more than 500 g of the CBB was placed in a preheated furnace at 700 °C for 30 min. The briquettes were weighed before and after preheating treatment. Then, the mass of the fines of CBBs over 0.5, 3.15 and 6.3 mm sieves was recorded. 

## 3. Results and Discussion

### 3.1. Binder Characterizations

**Figure 4:**
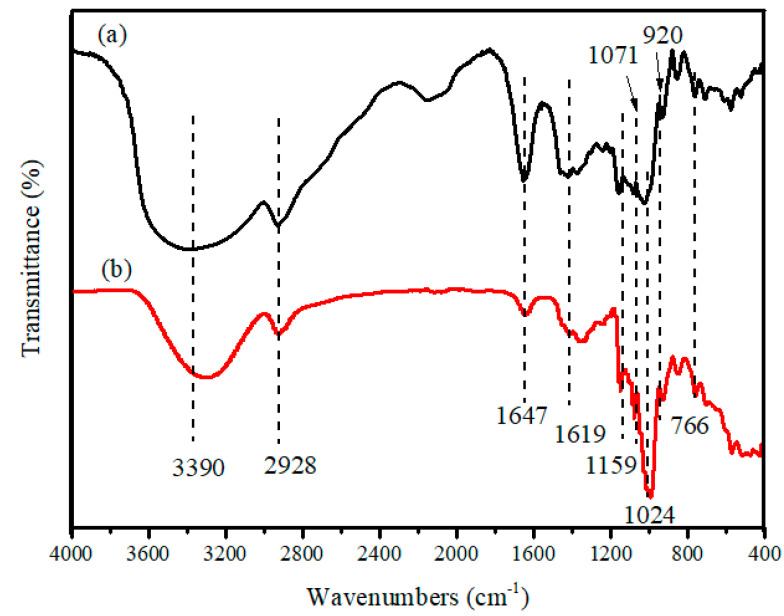
Fourier transform infrared (FTIR) spectroscopy spectra of binder (**a**) and corn starch (**b**).

**Figure 5:**
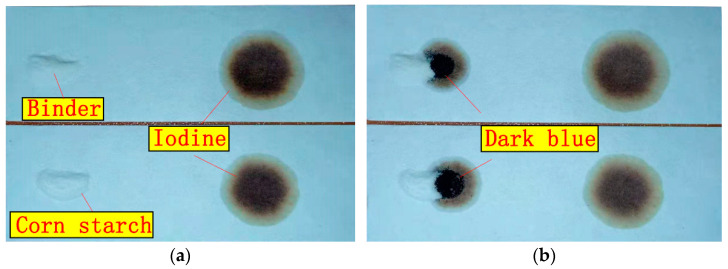
Experiment of binder and corn starch with dilute iodine solution. (**a**) Brilliant white of binder and corn starch; (**b**) iodine–binder and iodine-starch staining.

The results of organic element analysis of the binder are 39.85%, 6.08%, 53.94% and 0.13% for C, H, O and S, respectively. The results of XRF analysis of the binder ash are 48.45%, 50.54% and 0.05% for C, O and S, respectively. Therefore, these data indicate that the binder is an organic binder. In order to further understand the characteristics of the binder, its functional group was analyzed by FTIR spectroscopy. The FTIR spectra of the binder and corn starch are depicted in Figure 4. In the infrared spectrum of the binder, which is shown in Figure 4a, the extremely broad band at 3390 cm^−1^ and the band at 920 cm^−1^ are attributed to the O–H stretching. The band at 2928 cm^−1^ is attributed to the C–H stretching vibrations. The absence of the band at 1647 cm^−1^ is attributed to the H–O–H bending vibrations. The band at 1159 cm^−1^ is assigned to the C-O stretching vibrations. Moreover, the bands at 1071 and 1024 cm^−1^ are characteristic of the C–O–C stretching vibrations. According to the above band assignments [14,15,16], the band assignments of the binder matches that of corn starch, which is shown in Figure 4b. In addition, the binder was also proved to be a kind of starch by iodine-starch staining, as shown in Figure 5.

**Figure 6:**
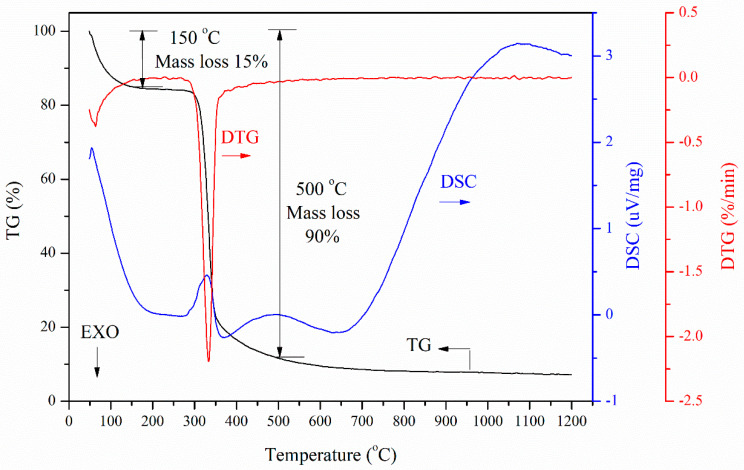
TG, DTG and DSC curves for binder during the heating process.

Binder has a great influence on the decrepitation temperature of the CBB [10]. The thermal properties of the binder were characterized by TG, DTG and DSC under N_2_ atmosphere. The thermograms of the binder are shown in Figure 6. From the TG curve, there are two temperature ranges with large mass loss. The mass loss reaches 15% at 150 °C and 90% at 500 °C. From the DTG plot, it can be observed that the maximum mass loss of the binder is near 340 °C. The mass loss due to the release of volatile matter at near 340 °C and complete release at 500 °C. Furthermore, mass loss of the binder remains stable after 500 °C, and the decomposition of the remaining 10% mass occurs at a very slow rate through the rest temperature range of treatment. There are four endothermic peaks in the DSC curve. The first endothermic peak at 180 °C is due to moisture desorption; the second endothermic peak at 276–361 °C and the third endothermic peak at 372–625 °C correspond to the ash volatilization and the pyrolysis of macromolecular organic matter, respectively. The last endothermic peak observed after 650 °C is caused by the decomposition of materials with high activation energy [17].

### 3.2. Relationship between Mechanical Properties and the Binder

**Figure 7:**
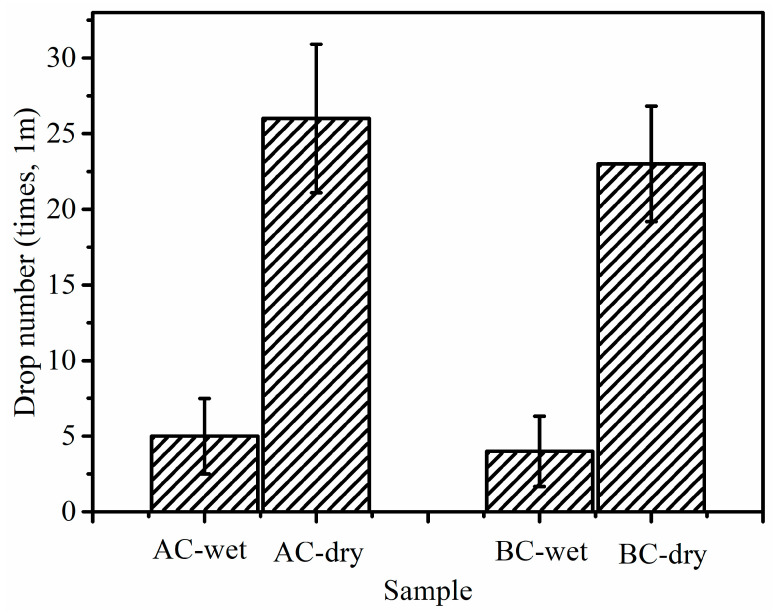
Dropping times of the wet and dry CBBs.

**Figure 8:**
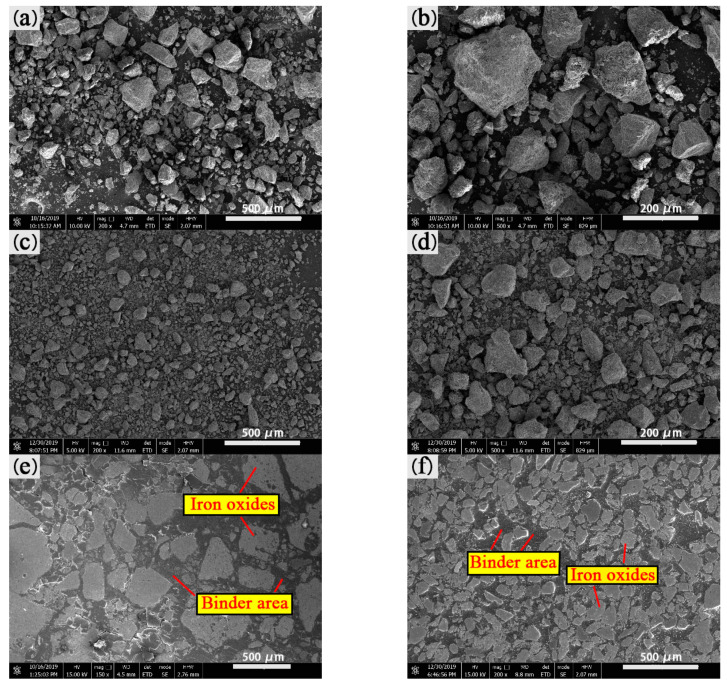
SEM images of sample A (**a**,**b**), sample B (**c**,**d**), sample AC (**e**) and sample BC (**f**).

CBBs with good strength can avoid cracking and generating fines during transportation. In the dropping examination, the samples were dropped onto a 10 mm-thick steel plate from 1 m height. The results of the drop examination are shown in Figure 7. The dropping times of dry AC and BC are longer than those of wet samples (Figure 7). This is due to the mixture of large and small particles in sample A (see Figure 8e) making the briquette stronger than uniform particle size in sample B (see Figure 8f) [18]. The microstructure of the two samples was investigated by SEM, Sample B in Figure 8c,d has more uniform particle distribution than sample A in Figure 8a,b.

**Figure 9:**
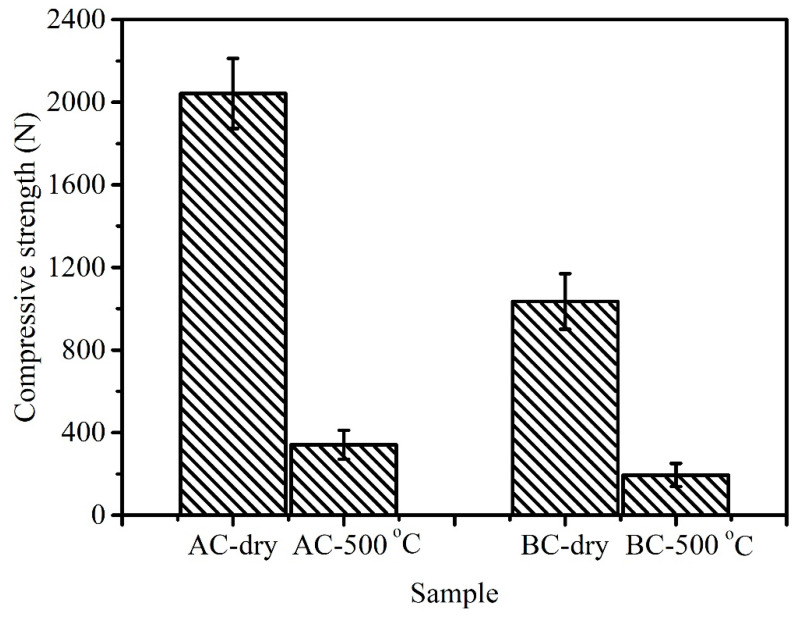
Compressive strength of the dry and heat-treated (at 500 °C for 30 min) CBBs.

The compressive strengths of samples AC and BC with dry and heat-treated at 500 °C conditions are shown in Figure 9. It can be seen that the compressive strength of the two samples decreases after 30 min at 500 °C in the muffle furnace (air atmosphere). The compressive strength of sample AC heat-treated at 500 °C can reach 341.62 N, which is 16.73% of the compressive strength value of dry sample AC. The compressive strength of sample BC heat-treated at 500 °C can reach 194.65 N, which is 18.80% of dry sample BC. The compressive strength values of heat-treated samples AC and BC are nearly one-fifth of dry samples AC and BC. In addition, it can be inferred from the information provided by Figure 6 that the binder still has a bonding effect after holding CBB at 500 °C for 30 min, while the remaining 10% mass [19], mechanical interlocking force, van der Waals bonding and electrostatic forces, etc., also contribute to the compressive strength of the CBB [20].

**Figure 10:**
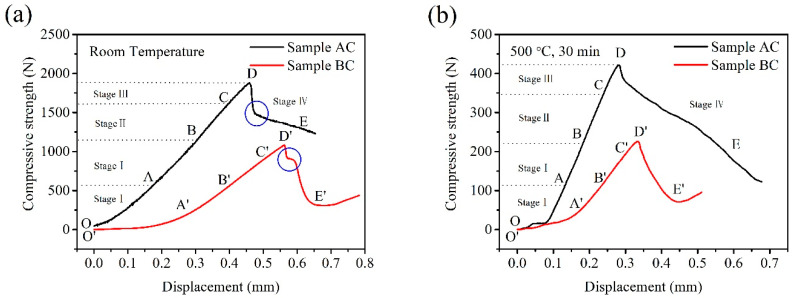
Typical compressive strength curve of CBB with dry (**a**) and heat-treated (**b**) at 500 °C for 30 min conditions.

**Figure 11:**
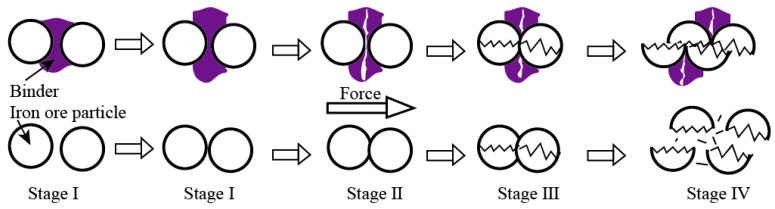
Diagram showing the development of the macroscopic failure process of dry sample (**top five**) and heat- treated sample (**bottom five**).

In order to better understand the change characteristics of compressive strength, typical compressive strength curves of two CBBs are shown in Figure 10. The change trend of compressive strength curves of sample AC before and after heat treatment at 500 °C are the same as those of sample BC. Both continuously increase during the process of applying compressive force and then decrease after reaching the peak. There are four characteristic stages in the development of the macroscopic failure process of intact CBBs: stage I: crack closure and elastic stage (OB), stage II: stable crack growth (BC), stage III: initiation of macro-scale shear failure (CD) and stage IV: instability propagation (DE) [21,22]. The difference between the dry and heat-treated sample during the macroscopic failure process is the instability propagation (DE) of stage IV. There is an obvious turning point (as shown in the blue circle in Figure 10a) on the curve; furthermore, there is even a transition platform on sample BC’s curve. However, there is no turning point (as shown in Figure 10b) on the curve. These findings suggest that the bonding strength of the binder decreases after heat treatment, and CBBs will produce more fine particles during the stages of instability propagation (DE). Figure 11 describes the development of the macroscopic failure process of the dry sample and heat-treated sample during the compressive strength test.

The temperature rises rapidly after the CBBs are charged in the blast furnace. The rapid change in temperature results in thermal stress of the CBBs, which encourages crack formation and makes the CBBs break up severely [23]. The fines of CBBs produced by the rise in temperature decrease the permeability of the bed and increase the dust content in stack gases. The decrepitation of the CBBs in the rapid heating process was evaluated by the decrepitation index. The results of the decrepitation index of samples AC and BC are shown in Table 3. It can be seen that samples AC and BC presented low decrepitation indices. Return fines of CBBs (sample AC) containing binder were applied in two blast furnaces in Northwest China. The industrial experiment results show that return fines of CBBs did not influence the performance of the blast furnace and can reduce the fuel rate to some degree. Table 4 shows the results of the effect of return fines of CBBs on the performance of two blast furnaces with different effective volume.

### 3.3. Microstructure and Bonding Mechanism

**Figure 12:**
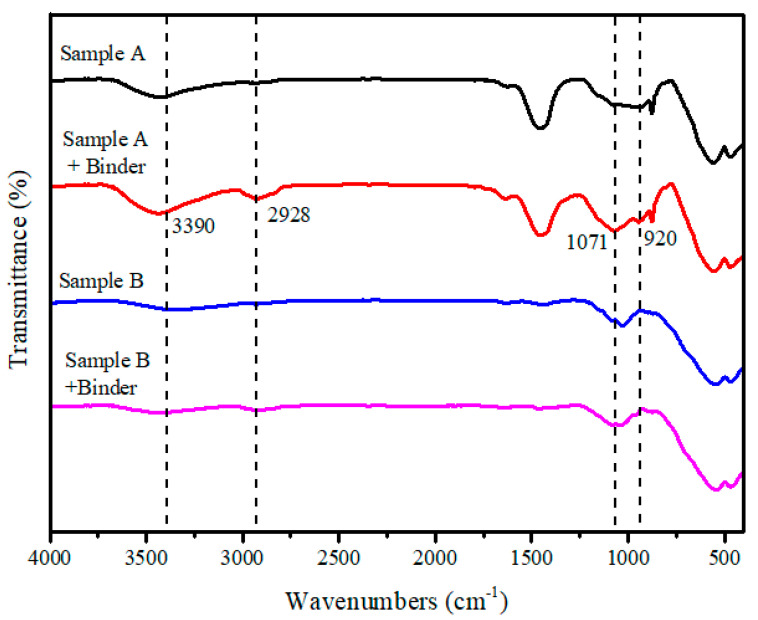
The infrared spectrogram of samples A and B with the binder.

The information about the structure and the nature of the functional groups present in the crystal lattice can be provided by FTIR [24]. An infrared spectrogram of samples A and B with the binder is shown in Figure 12. It can be concluded that chemisorption exists in sample A and does not exist in sample B after adding binder. In the curve of sample A, the characteristic peaks of the binder, e.g., O–H at 3390 and 920 cm^−1^, C–H at 2928 cm^−1^ and C–O–C at 1071 cm^−1^, can be detected on the surface of sample A + binder.

**Figure 13:**
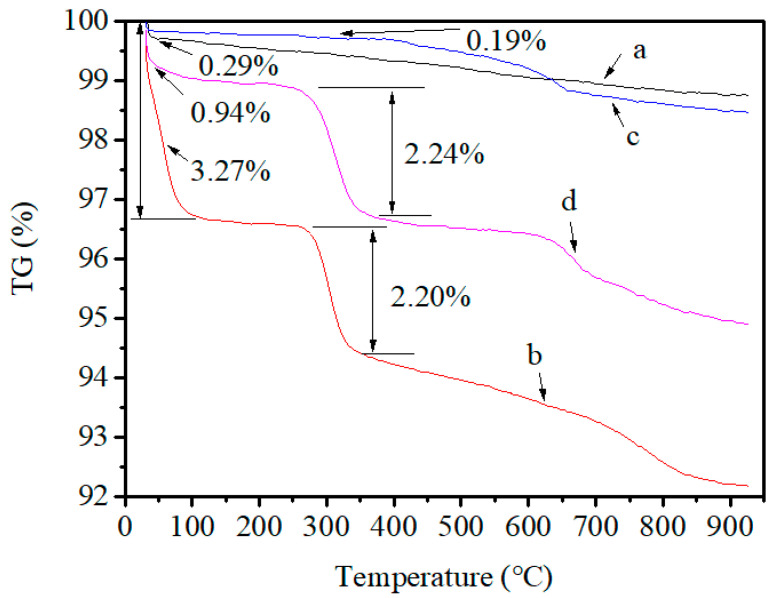
TG curves for (**a**) sample A; (**b**) the mixture of sample A, binder and water; (**c**) sample B; (**d**) the mixture of sample B, binder and water during the heating process.

Thermogravimetry (TG) was used to study the thermal behavior of sample A; the mixture of sample A, binder and water; sample B and the mixture of sample B, binder and water. The results of the thermal analyses are shown in Figure 13. The TG curves indicated that four curves have a similar thermal behavior in the temperature range below 100 °C, and the mass loss is related to the evaporation of physically adsorbed water. Figure 13a–d shows mass loss of 0.29%, 3.27%, 0.19% and 0.94%, respectively. The mass loss in b is higher than d, which indicates that b contains more water than d, which may aid bonding. Mass loss at around 300 °C in b and d is due to the binder decomposition and is similar, with values of 2.20% and 2.24%, respectively.

**Figure 14:**
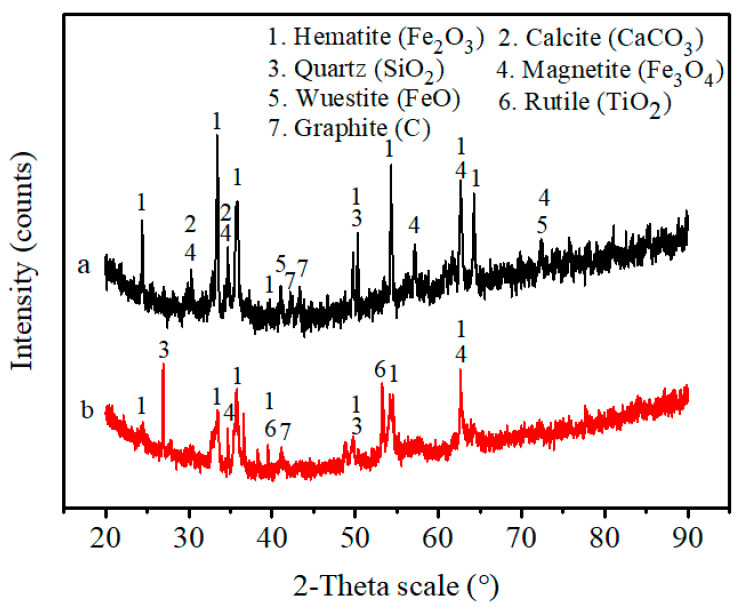
X-ray diffraction patterns for (**a**) the mixture of sample A, binder and water; (**b**) the mixture of sample B, binder and water.

The results for the mineralogical analysis of the mixtures are shown in Figure 14. The main crystalline phases of the mixtures of sample A, binder and water are hematite (Fe_2_O_3_), calcite (CaCO_3_), quartz (SiO_2_), magnetite (Fe_3_O_4_), wuestite (FeO) and graphite (C). The main crystalline phases of the mixtures of sample B, binder and water are hematite (Fe_2_O_3_), quartz (SiO_2_), magnetite (Fe_3_O_4_), rutile (TiO_2_) and graphite (C). Comparing with the results shown in Figure 1, the phase of graphite (C) is present in the results shown in Figure 14 due to the mixture containing binder.

**Figure 15:**
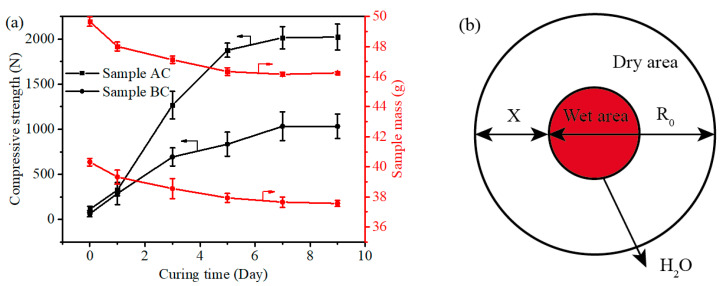
Evolution of compressive strength and the mass of samples AC and BC during the curing process (**a**) and the model of the curing process (**b**).

The effect of the binder on the curing process of samples AC and BC was studied, and the results are shown in Figure 15. Figure 15a shows the evolution of compressive strength and the mass of samples AC and BC while drying in the air during the curing process. The compressive strength of the CBBs increases obviously with the increase of curing time because the gluing effect of the binder increases steadily, and the mass of the samples decrease continually due to the evaporation of moisture. Before the fifth day of the curing process, the moisture evaporation and compressive strength rapidly increased. The weight loss and strength remained almost constant after the seventh day, which indicates that the curing time must be controlled in 7 days.

The curing rate of the binder can be calculated by the model of solid-phase reactions. It is assumed that the curing process proceeds from the surface of the sphere to the center and that the diffusion at the cross-sectional area is constant. The schematic of the model is shown in Figure 15b. The curing process of the binder is simplified as a process with decreasing rate; curing rate is related to the moisture capacity:

(1) $$\frac{dw}{Fdt}=k_{c}\left(C-C_{E}\right)$$

where $\frac{dw}{Fdt}$ is curing rate of binder, $k_{c}$ is scale factor, *C* is moisture capacity of the CBB at time *t* and *C_E_* is equilibrium moisture content of the CBB. The volume ratio of the remaining dried CBB at time t is expressed by R:

(2) $$R={\left(\frac{R_{O}-X}{R_{0}}\right)}^{3}$$

where *R*_0_ is an equal diameter pellet of wet area and *X* is the layer thickness of dry area at time t. The parabolic equation of the solid-phase reaction is as follows:

(3) $$X^{2}=\mathrm{Kt}$$

where K is scale factor of the solid-phase reaction. *C*–*C_E_* can be expressed by R; therefore, substituting Equations (2) and (3) into Equation (1), Equation (4) is obtained:

(4) $$\frac{dw}{Fdt}=k_{c}{\left(\frac{R_{O}-X}{R_{0}}\right)}^{3}=k_{c}{\left(\frac{R_{O}-\sqrt{kt}}{R_{0}}\right)}^{3}$$

$R_{0}\geq \left(R_{O}-\sqrt{kt}\right)$; therefore, curing rate of the binder decreases and the rate of compressive strength decreases during the curing process.

**Figure 16:**
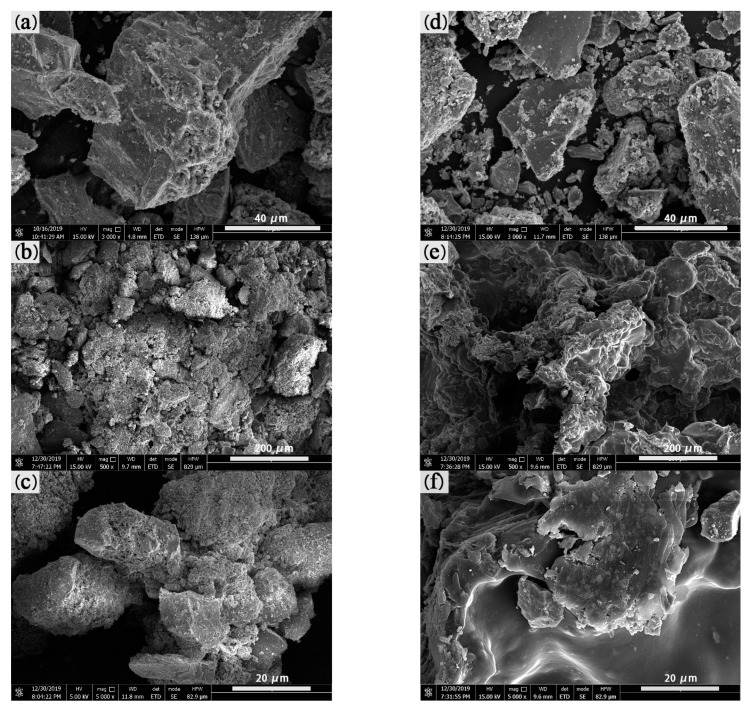
SEM images of (**a**) iron ore particles of sample A; (**b**,**c**) the mixture of sample A, binder and water; (**d**) iron ore particles of sample B; (**e**,**f**) the mixture of sample B, binder and water.

Investigating the microstructure of the CBB helps to understand the bonding mechanism. The SEM images of the samples including samples A, B and their mixtures with binder and water were examined to investigate the microstructure of the briquettes before and after adding the binder, as shown in Figure 16. As seen in Figure 8a,b and Figure 16a, fines of sample A without adding binder exist in the form of a single particle and have sharp edges and corners, and the surface is fully filled by the bumps and the holes. After mixing with water and binder, as shown in Figure 16b, the surfaces of iron ore particles are covered with the binder film. Iron ore fines in sample A are glued together and exist in the form of aggregation. The edges and corners of the fines are obscure, and the surfaces of coarse particles are covered with small particles as shown in Figure 16c. Therefore, as shown in Figure 16a–c, the connection among different-sized particles become closer.

The microstructure characteristics of sample B shown in Figure 16d resemble those of Figure 16a. Iron ore particles have individual rough surfaces with sharp edges and corners. As shown in Figure 16e, iron ore particles bonding together exist in the shape of a cluster, the surface of which is smooth, and the original surface is completely covered with binder film. There is a large amount of film that is formed by binder hydration on the surface of the iron ore particles as shown in Figure 16f. Therefore, it is difficult to distinguish the edges and corners of iron ore particles. Compared with the microstructure of sample A after adding the binder, the surface of sample B is completely covered with the binder film, while sample A has a thinner film layer, which could be the reason for the transition platform present on sample BC’s compressive strength curve as shown in Figure 10a.

**Figure 17:**
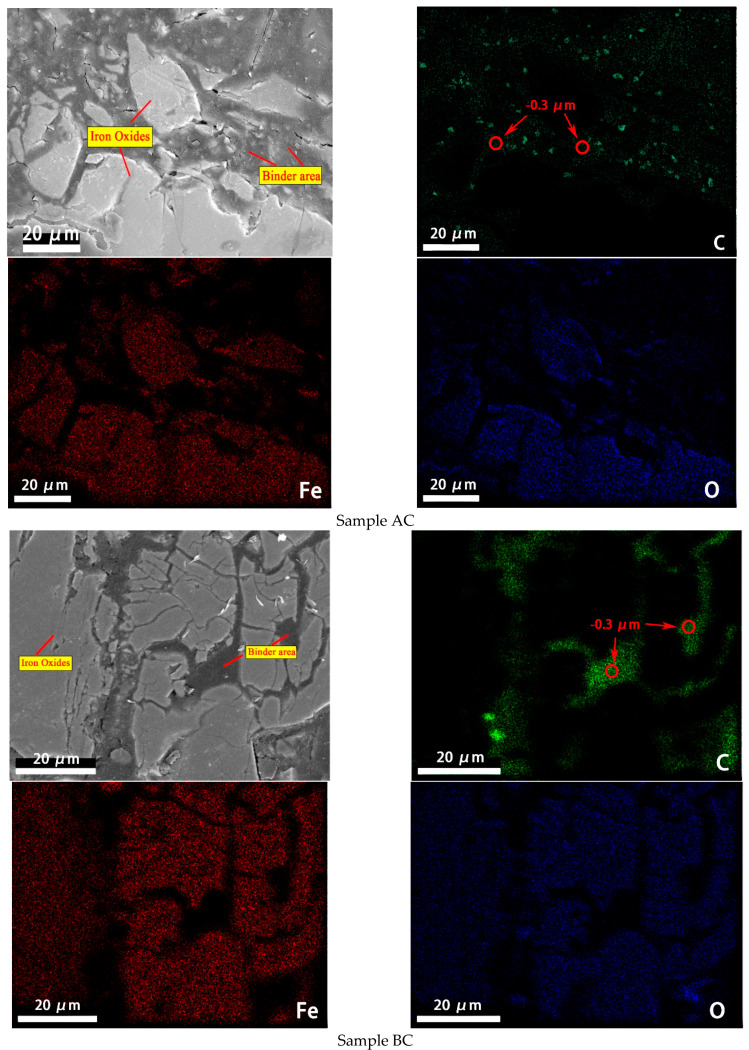
Elemental map of sample AC (**top four**) and BC (**bottom four**) determined by EDS analysis.

**Figure 18:**
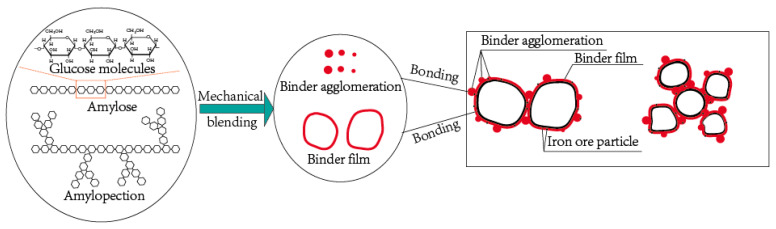
Schematic diagram of the binder bonding the iron particles through binder film and binder agglomeration.

The microstructure and microanalyses of samples AC and BC were obtained by means of SEM-EDS. Figure 17 is an EDS surface scan analysis of samples AC and BC. It can be clearly seen that carbon is mainly distributed around the iron oxide area. There is aggregation of carbon in sample AC and to a lesser extent in sample BC. Combined results of element analysis are presented in Table 2. The carbon in aggregation in sample AC is from raw material due to sample A containing carbon. Because no carbon was found in the raw material of sample BC, carbon in sample BC reflects the distribution of the binder. Moreover, the size of carbon particles in sample BC was less than 0.3 μm. Therefore, it can be inferred that carbon particles with 0.3 μm size in sample AC come from the binder. Carbon particles with 0.3 μm size in samples AC and BC indicate the binder agglomeration and nonuniform distribution. However, binder film covers the particles as shown in Figure 16, and the carbon content is too low to be detected by EDS. The binder exists in film and agglomeration form, and these two forms work together to increase the CBB’s strength. The working mechanism of the binder is summarized in Figure 18. Starch consists of amylose and amylopectin, the normal ratio of which is 27:73%, and the binding power of starch appears to be provided by the amylopectin fraction [25]. The multi-branched structure of amylopectin provides omnibearing adhesion sites [26], thus forming binder agglomeration and film and leading to a strong adhesion between binder and iron ore particles.

## 4. Conclusions

The present work investigated the effect of an organic binder on the properties of CBBs prepared from iron ore fines. The main conclusions of the current study can be summarized as follows:

1. The chemical composition, iodine-starch staining and Fourier transform infrared analyses indicated that the binder was a type of starch. During the heating procedure, the mass loss of the binder reached 15% at 150 °C and 90% at 500 °C. The binder pyrolysis underwent four stages: moisture desorption, ash volatilization, pyrolysis of macromolecular organic matter and decomposition of materials with high activation energy when heated.
2. The compressive strength values of heat-treated CBBs are nearly one-fifth of the compressive strength values of the dry CBBs. The difference between the dry and heat-treated sample was owing to the instability propagation of the crack. Return fines of CBBs containing binder were applied in the two blast furnaces. The industrial experiment results show that return fines of CBBs did not influence the performance of the blast furnace and can reduce the fuel rate to some degree.
3. The curing rate of the binder decreases and the rate of compressive strength decreases during the curing process. The edges and corners of the particles become blurred, and the original surface of the particles are covered with binder film, the surface of which is covered with fine particles. The multi-branched structure of amylopectin provides omnibearing adhesion sites, thus forming binder agglomeration and film leading to a strong adhesion between binder and iron ore particles.

## Figures and Tables

**Table 1:** Particle size distribution of samples A and B (wt.%).

Particle Size Range	<0.5 mm	0.5–2 mm	2–3.15 mm	3.15–5 mm
Sample A	19.56	38.42	15.24	26.68
Sample B	100	0	0	0

**Table 2:** Chemical composition of iron ore raw material (wt.%).

Constituents	Sample A	Sample B
Fe (total)	58.36	56.12
FeO	7.46	0.41
SiO_2_	6.51	2.76
CaO	8.11	0.11
Al_2_O_3_	1.19	1.36
MgO	1.27	0.07
TiO_2_	-	15.28
MnO	0.31	0.26
S	0.05	0.02
C	2.39	-

**Table 3:** The decrepitation index of samples AC and BC.

The Decrepitation Index	Sample AC	Sample BC
Mass of dry sample before treatment, g	544.1	551.04
Mass of sample after treatment at 700 °C for 30 min, g	523.9	519.29
Mass of sample pass 0.5 mm after treatment, g	0.55	0.57
Mass of sample pass 3.15 mm after treatment, g	0.89	2.05
Mass of sample pass 6.3 mm after treatment, g	0.89	2.05
Loss on ignition, %	3.71	5.76

**Table 4:** Effect of return fines of CBBs on the performance of two blast furnaces with different effective volume.

Effective Volume of Blast Furnace (m^3^)	Weight Percentage of CBB (%)	Productivity (t/(m^3^·day))	Coke Rate (kg/tHM)	Coal Rate (kg/tHM)	Fuel Rate (kg/tHM)
450	0%	3.32	424.32	143.21	538.89
	3%	3.36	417.93	145.69	534.48
	6%	3.42	419.23	137.82	529.72
	9%	3.45	417.46	149.39	536.97
2800	0%	2.26	443.73	102.38	525.63
	1.5%	2.37	437.58	106.00	522.38

## Data Availability

Data are contained within the article and can be requested from the corresponding author.
